# Alginate Beads with Encapsulated Date Palm Pollen Extract: Development, Characterization and Their Potential Role in Hepato-Protection and Fertility-Stimulating Hormones Improvement in Bisphenol A-Treated Rats

**DOI:** 10.3390/polym17070912

**Published:** 2025-03-28

**Authors:** Karem Fouda, Rasha S. Mohamed

**Affiliations:** Department of Nutrition and Food Sciences, National Research Centre, Dokki, Cairo 12622, Egypt; karemfouda@gmail.com

**Keywords:** beads, alginate, encapsulation, date palm pollen, phenolic compounds, bisphenol A

## Abstract

The goal of this study was to design polymeric beads with a core of date palm pollen (DPP, *Phoenix dactylifera* L.) extract using the ionic gelation method and then assess the effects of the extract in combination with alginate polymer (alginate/DPP beads) on the profile of phenolic compounds, their in vitro controlled release, as well as their antioxidant characteristics, and potential role in hepato-protection and fertility-stimulating hormones improvement in bisphenol A (BPA)-treated rats. The encapsulation efficiency (EE) was 94.27 ± 1.47%. The study found that phenolic release was highest (89.81%) at pH 7.4 (simulated intestinal fluid) and lowest (59.43%) at pH 2 (simulated stomach fluid) after 3 h. This particular type of bead also exhibited significant antioxidant activity, phenolic component content, and flavonoid content. The estimated phenolic content was 66.48 mg GAE/g, with methyl gallate, gallic acid, and naringenin as the main components. In vivo evaluation findings revealed that both doses of alginate/DPP beads (delivering 250 and 350 mg/kg of extract per day) significantly protected the liver (as demonstrated by downregulated liver function parameters), improved levels of male fertility-stimulating hormones, reduced oxidative stress parameters and inflammatory cytokines, and protected both liver and testicular tissues from BPA-induced changes. Thus, the actions of alginate/DPP beads make them a promising choice for antioxidant, liver-protecting, and male hormone-enhancing hydrogels.

## 1. Introduction

Polymeric beads have recently received a lot of attention due to their adaptability to a variety of biological applications. Numerous substances can be enclosed in these beads, protecting them from degradation and allowing for controlled release. These beads improve solubility, stability, and bioavailability by encapsulating therapeutic ingredients, such as physiologically active components or bioactive compounds. This could potentially improve the therapeutic efficacy and selectivity [1]. Alginate, isolated from brown seaweed, is a linear polyanionic polysaccharide composed of (1–4)-linked β-D-mannuronate and its C5-epimer, α-L-guluronate [2]. Alginates are widely used in the food, medical, and cosmetic industries due to their biocompatibility and biodegradability. Encapsulated spheres with alginates have a high ability to integrate and release bioactive compounds [3]. This aroused our interest in using alginate to prepare beads of bioactive compounds extracted from date palm pollen to preserve these bioactive compounds and also to mask the unpalatable taste of these compounds.

Date palm pollen (DPP, *Phoenix dactylifera* L.), a fine powder produced by pollen grains, is used in traditional medicine because it contains many antioxidant and antibacterial phytochemicals in addition to the nutritional value represented by the presence of protein, minerals, dietary fiber, vitamins, sugars, amino acids, lipids, hormones, carbohydrates, and sterols [4]. Al-Asmari et al. [5] discovered that in experimental rats given acetaminophe, DPP prevented oxidative damage to the hepatic and renal tissue. Al-Asmari et al. [5] came to the conclusion that DPP’s protective function might result from its anti-hyperlipidemic, membrane-stabilizing, and antioxidant properties, which are achieved via modifying biochemical indicators. The potent and advantageous components of DPP, such as phenolics, flavonoids, carotenoids, and other nutrients, may be responsible for its broad spectrum of pharmacological activities. Additionally, DPP extracts contain components that ameliorate male infertility and increase gonadotropin activity [6]. According to Salhi et al. [6], numerous studies have shown that aqueous and ethanolic extracts of DPP, which contain polar compounds, particularly phenols and flavonoids, improve testicular health, hormone levels, and sexual behavior. These compounds are known to have strong biological effects and improve reproductive health indicators because of their affinity for cellular components, including enzymes, receptors, and signaling pathways necessary for reproduction. Furthermore, by lowering oxidative stress and scavenging free radicals via H-atom transfer caused by lipid peroxidation, flavonoids and phenolic compounds’ antioxidant properties may help preserve cell membranes and their contents from oxidative damage. Given that it contains carotenoids, tannins, saponins, flavonoids, and steroidal substances like estrogen, cholesterol, estrone, estradiol, and sterol, and is regarded as a potent booster of sexual potency and fertility [7]. These properties led us to investigate whether DPP could protect and prevent the harmful effects of some of the compounds to which we are exposed, causing liver problems or harming fertility, the most famous and widespread of which is bisphenol A (BPA). BPA is a highly manufactured chemical that is widely utilized in a variety of consumer products, including polycarbonate plastics, epoxy resins, PVC, food packaging, dental sealants, and thermal paper receipts, and migrates from cans and packaging to food [8]. BPA toxicity is manifested by lipid peroxidation and the formation of free radicals, which cause oxidative stress and may result in liver malfunction and reproductive damage [9]. BPA can bind to the androgen receptor as an antagonist, disrupting the hypothalamic-pituitary-testicular axis and influencing testicular steroidogenesis enzymatic activity and gene expression, which can result in hypogonadotropic hypogonadism [10].

The objectives of this study were to develop and characterize alginate/DPP extract beads and investigate their potential role, compared to DPP powder, in hepato-protection, fertility-stimulating hormone improvement, and safeguarding testicular and hepatic tissues from the detrimental effects of BPA.

## 2. Materials and Methods

### 2.1. Materials

Date palm pollen powder was purchased from a local market (Haraz, Cairo, Egypt). Sodium alginate (medium viscosity, 240–3500 mPa·s) was purchased from Loba Chemie, Pvt Ltd. in Mumbai, India. 2,2-diphenyl-1-picrylhydrazyl (DPPH), and Folin–Ciocualteu phenol reagent were purchased from Sigma-Aldrich (St. Louis, MO, USA). All other chemical reagents and solvents were of high analytical grade.

### 2.2. Methods

#### 2.2.1. Preparation of DPP Extract

According to El-Neweshy et al. [11], 5 g of DPP powder was sonicated in 100 mL of ethanol (70% *v*/*v*) for 30 min at 250 watts of power and 20 kHz using an ultrasonic probe (vibra cell; Sonics & Materials, Inc., Newtown, CT, USA). Three identical extraction methods were used. To avoid overheating, the extraction beaker was placed in an ice bath. Following centrifugation at 12,000× *g* for 30 min, the supernatants from the three extractions were mixed. A rotary evaporator (BÜCHI Labortechnik AG, Flawil, Switzerland) was used to evaporate the solvents. The residue was stored at −18 °C after drying for 48 h at 1.03 mbar in a freeze dryer (ALPHA 1–2 LD plus, Osterode, Germany) at −52 °C.

#### 2.2.2. Preparation of Alginate/DPP Extract Solution

Sodium alginate (3% *w*/*v*) was mixed with distilled water and agitated repeatedly to generate a homogeneous solution. The DPP extract (5% dry) was spun magnetically for 5 min. The mixture was homogenized at 10,000 rpm for 1 min.

#### 2.2.3. Measuring of Particle Size Distribution and Zeta Potential of Alginate/DPP Extract Solution

Using a particle sizing equipment (Particle Sizing Systems Corporate, Santa Barbara, CA, USA), dynamic light scattering (DSL) was employed to determine the particle size distribution and zeta potential. A polystyrene cell was used to determine the particle size. With a wavelength of 633 nm and a detector angle of 90°, the zeta potential was measured in a zeta cell at room temperature. Prior to the analysis, the solution was diluted with distilled water to a suitable concentration (1:10 *v*/*v*) to prevent the effect of multiple scattering.

#### 2.2.4. Preparation of Alginate/DPP Extract Beads

**Figure 1:**
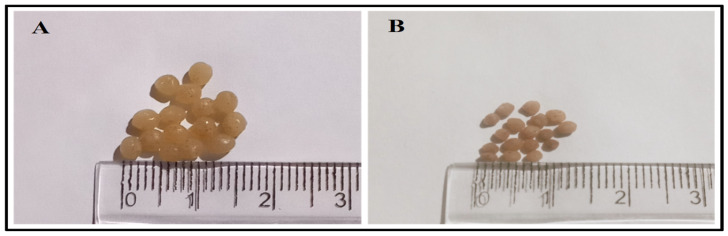
Photographs of the wet alginate/DPP beads (**A**) and the dried alginate/DPP beads (**B**) (Magnification 4×).

The ionic gelation method was used to develop the alginate/DPP beads [12]; a syringe with a 0.5 cm gauge needle was used to transfer the solution into a CaCl_2_ solution (2% *w*/*v*) to develop sphere-shaped alginate/DPP beads. Furthermore, there was a 5 cm gap between the tip of the needle and the CaCl_2_ solution. Three rounds of rinsing and filtering were performed on the spherical beads using distilled water. The beads (Figure 1) were air-dried before being stored in a refrigerator until use.

#### 2.2.5. Encapsulation Efficiency (EE)

The beads (45 mg) were suspended in 5 mL of water and agitated for 10 min using the protocol devised by Siles-Sánchez et al. [13] to measure the efficacy of encapsulation. The supernatant (500 µL) containing non-encapsulated components was centrifuged at 3000× *g* for 15 min. The amount of non-encapsulated extract was measured by determining the non-encapsulated total phenolic content using the Folin-Ciocalteu technique, which will be described in detail later.

The following equation was used to calculate the EE%:

EE (%) = 100 − (sum of the supernatant phenolic compounds/sum of the phenolic compounds in extract) × 100(1)

#### 2.2.6. Scanning Electron Microscopy (SEM)

The shapes of the DPP grains and the beads of alginate/DPP extract were studied using a scanning electron microscope TESCAN VEGA 3 (Tescan, Brno, Czech Republic). The samples were attached to a metal support with a double-sided adhesive and carbon-coated with a thin gold layer. Visualization was carried out using a 20 kV excitation voltage at different magnifications.

#### 2.2.7. Extraction Procedure of the Alginate/DPP Beads

Ten mL Ten mL of 70% ethanol was used to suspend three grams of the beads. After homogenizing the samples, they were ultrasonically extracted for 30 min at 30 °C. Following this, the extracts were centrifuged, and analyses were conducted using the supernatants that were produced.

#### 2.2.8. High Performance Liquid Chromatography (HPLC) of the Beads Extract

HPLC analysis was carried out using an Agilent 1260 series instrument. For separation, an Eclipse C18 column (4.6 mm × 250 mm i.d., 5 m) was used. The mobile phase was composed of water (A) and 0.05% trifluoroacetic acid in acetonitrile (B) at a flow rate of 0.9 mL/min. The mobile phase was designed using the following linear gradient sequence: 0 min (82% A), 0–5 min (80% A), 5–8 min (60% A), 8–12 min (60% A), 12–15 min (82% A), 15–16 min (82% A), and 16–20 min (82% A). The multi-wavelength detector was set at 280 nm. The injection volume of each sample solution was 5 µL. The temperature of the column was maintained at 40 °C. The concentration of each compound was determined by comparing the peak area of the sample to that of the standard (gallic acid, chlorogenic acid, catechin, methyl gallate, coffeeic acid, syringic acid, pyrocatechol, rutin, coumaric acid, vanillin, ferulic acid, naringenin, daidzein, quercetin, cinnamic acid, apigenin, kaempferol, and hesperetin).

#### 2.2.9. Determination of the Total Phenolic Content (TPC) of the Beads Extract

With some slight modifications, the Folin-Ciocalteu technique [14] was used to calculate the total phenolic content. The extract (50 μL) was mixed with 250 μL of Folin-Ciocalteau reagent, and the volume was adjusted to 3.5 mL with distilled water. After five minutes, 0.5 mL of a 20% aqueous sodium carbonate (NaCO_3_) solution was added to the liquid to neutralize it. The absorbance was measured at 765 nm with respect to the solvent blank after 40 min. The total phenolic content was calculated using a calibration curve made using gallic acid and is represented as mg of gallic acid equivalent per gram of extract (mg GAE/g extract).

#### 2.2.10. Determination of the Total Flavonoid Content (TFC) of the Beads Extract

The total flavonoid concentration was measured using the aluminum chloride (AICI_3_) colorimetric test, as described by Mare et al. [14]. In summary, 50 µL of the extract was combined with 300 µL of 5% sodium nitrite (NaNO_2_). After incubation for 6 min, 300 μL of a 10% AICI3 solution was added, and distilled water was added to adjust the total volume to 1.80 mL. 1.5 mL of 1 M NaOH was added to the mixture. The absorbance of the supernatant was measured at 420 nm in relation to the solvent blank. The total flavonoid concentration was calculated using a calibration curve created using catechin and is represented as milligrams of catechin equivalent per gram of extract (mg CE/g extract).

#### 2.2.11. In Vitro Determination of Antioxidant Activity of the Beads Extract

The stock solution was successively diluted and used for in vitro assays. Sample concentrations (mg mL^−1^) that resulted in 50% antioxidant activity or 0.5 absorbance (EC_50_) were calculated from graphs of antioxidant activity percentages (DPPH and TBARS tests).

##### DPPH Radical Scavenging Activity Assay

The procedure was carried out using an ELX800IU-N microplate reader (Bio-Tek Instruments, Inc.; Winooski, VT, USA) in accordance with Corrêa et al. [13]. The reaction mixtures in the 96-well plate included a methanolic solution (270 μL) containing DPPH radicals (6 × 10^−5^ mol L^−1^) and extract solutions of varying concentrations (30 μL). The absorbance at 515 nm was measured after each mixture was allowed to stand in the dark for 30 min. The following equation was used to determine the radical scavenging activity (RSA) as a percentage of DPPH discoloration:

%RSA = [(ADPPH − AS)/ADPPH] × 100(2)

where AS is the absorbance of the solution containing the sample and ADPPH is the absorbance of the DPPH solution.

##### Thiobarbituric Acid Reactive Substances (TBARS) Assay

With a small modification, this assay was performed as described by Corrêa et al. [15]. Egg homogenate (100 μL, 10% in distilled water, *v*/*v*) was incubated with various concentrations of sample solutions (200 μL) in the presence of FeSO_4_ (10 mM; 100 μL) and ascorbic acid (0.1 mM; 100 μL) at 37 °C for one hour. The reaction was obstructed by adding trichloroacetic acid (28% *w*/*v*, 500 μL) and thiobarbituric acid (TBA, 2%, *w*/*v*, 380 μL). The mixture was then heated at 80 °C for 20 min, and the precipitated protein was removed by centrifugation at 3000× *g* for 10 min. The color intensity of the malondialdehyde (MDA)-TBA complex in the supernatant was determined at an absorbance of 532 nm. The inhibition ratio (%) was calculated using the following formula:

Inhibition ratio (%) = [(A − B)/A] × 100%(3)

where A and B are the absorbances of the control and sample solutions, respectively.

#### 2.2.12. Assessment of the Controlled Release of Phenolic Compounds from the Beads

With a few minor modifications, the release of phenolic compounds was investigated using the methodology described by Siles-Sánchez et al. [13]. The particles (45 mg) were suspended in 5 mL of acetate buffer with pH 2 and phosphate-buffered saline (PBS) solution with pH 7.4. The suspensions were submerged in a water bath at 37 °C and stirred. At 1, 2, and 3 h, 350 µL of solution was removed from each tube and centrifuged at 3000× *g* for 15 min. After collecting the supernatants, the release of phenolic compounds at each time point was calculated.

#### 2.2.13. In Vivo Assay

##### Animals and Experimental Design

Thirty adult male Wistar rats with an average weight of 168.4 ± 6.5 g (mean ± SD) were obtained from the animal house of the National Research Centre, Egypt. The animals were maintained under standard laboratory conditions of humidity (55–60%) and temperature (22 ± 2 °C), and a 12-h light/dark cycle (light from 7:00 to 19:00) with free access to food and tap water. The animals were individually housed in cages and fed a maintenance standard diet prepared according to Reeves et al. [16] containing 12% protein, 10% corn oil, 10% sucrose, 58.5% starch, 5% fiber, 3.5% AIN-93 salt mixture, and 1% AIN-93 vitamin mixture. The study protocol was approved by the National Research Center’s Medical Research Ethics Committee (MREC) with Ethical Approval Certificate No. 34912012023 and was conducted in accordance with the NIH guidelines.

Following acclimatization for one week, the rats were randomly divided into five groups (n = 6 per group) as follows: Group 1 (CN): Rats in the control group that were treated with corn oil only (0.2 mL/day; P.O.). Group 2 (BPA): Rats were treated with bisphenol A (30 mg/kg/day; P.O. dissolved in 0.2 mL of corn oil). Group 3 (DPP): Rats were treated with bisphenol A (30 mg/kg/day; P.O. dissolved in 0.2 mL of corn oil) and co-treated with DPP powder (100 mg/kg/day; suspended in 2 mL distilled water). Group 4 (LDPPE): Rats were treated with bisphenol A (30 mg/kg/day; P.O. dissolved in 0.2 mL of corn oil) and co-treated with a low dose of DPP extract (250 mg/kg/day) presented in 5 g beads. Group 5 (HDPPE): Rats were treated with bisphenol A (30 mg/kg/day; P.O. dissolved in 0.2 mL of corn oil) and co-treated with a high dose of DPP extract (350 mg/kg/day) presented in 7 g beads. All treatments were administered for 30 consecutive days. BPA dose selection was performed according to Liu et al. [8]. The concentrations of DPP powder and extract were determined based on the findings of a previous study conducted by Al-Asmari et al. [5] and Hajb et al. [17]. At nine a.m., before feeding the animals the balanced diet, the beads were delivered in a clean crockery mixed with 5 g of the balanced diet to ensure that each rat consumed the required quantity. Food intake was recorded on a daily basis during the experiment. The investigation concluded with the calculation of the total food intake, body weight gain, and feed efficiency ratio. Following an overnight fast, blood samples were collected from rats that had been gently anesthetized. The blood samples were centrifuged at 3500 rpm for 15 min to extract serum, which was then stored at −80 °C until analysis. Finally, the rats were euthanized by decapitation and dissected to obtain the liver and testes.

##### Biochemical Analysis

Serum from each rat was analyzed for testosterone (T), luteinizing hormone (LH), follicle-stimulating hormone (FSH), estradiol, C-reactive protein (CRP), interlukin-6 (IL-6), and tumor necrosis factor (TNF-α) using sandwich ELIZA detection kits (Sunlong Biotech, Hangzhou, Zhejiang, China) and the above mentioned microplate reader. The levels of total and direct bilirubin were determined according to Balistreri and Shaw [18]. The activities of alkaline phosphatase (ALP), aspartate transaminase (AST), alanine transaminase (ALT), and gamma-GT (γ-GT) were determined according to the method described by Bessey et al. [19], Reitman and Frankel [20], and Szasz [21]. The levels of albumin, creatinine, and urea and the activity of lactate dehydrogenase (LDH) were determined according to Doumas et al. [22], Larsen [23] and Fawcett and Scott [24] and Zimmerman and Weinstein [25], respectively.

##### Oxidative Stress Markers

Red blood cell reactive oxygen species (ROS) levels were determined using a sandwich ELISA detection kit (SinoGeneclon Biotech Co., Ltd., Hangzhou, China) and the above mentioned microplate reader. Red blood cell lipid peroxidation (MDA), glutathione peroxidase (GPx), nitric oxide (NO), and superoxide dismutase (SOD) activities were determined according to Ohkawa et al. [26], Paglia and Valentine [27], Montgomery and Dymock [28], and Nishikimi et al. [29], respectively.

##### Histopathological Examination

According to Bancroft and Cook [30], the liver and test tissues of the various experimental groups were fixed in 10% formal saline. The tissues were then processed for pathological examination using an automatic tissue processor and sectioned using a rotary microtome to a thickness of 5 μm. The sections were stained with haematoxylin and eosin (H&E) and evaluated under a Nikon research microscope (Nikon Ti-Eclipse, equipped with an ORCA-4.0s CMOS camera, Hamamatsu) for analysis.

##### Statistical Analysis

The data from the animal experiments are presented as mean ± standard error (SE) and were statistically analyzed using one-way analysis of variance (ANOVA) in SPSS version 21. Statistical differences across groups were analyzed using Duncan’s test, with a difference considered statistically significant at *p* ≤ 0.05.

## 3. Results and Discussion

To preserve and benefit from the phytochemical characteristics of compounds derived from plants, it is suggested that bioactive compounds be encapsulated in a protective matrix. By preventing degradation from external elements like light, oxygen, or pH, encapsulation extends the product’s shelf life and enables the development of substances for the food and pharmaceutical industries that may retain their bioactivity for a prolonged period of time [31]. Alginate is a natural carbohydrate often utilized in the microfluidics process to develop porous polymeric beads. Because of their large surface area, low density, high surface-penetrating capacity, and good stability, beads with internal or external pores offer space for the incorporation and controlled release of medications, bioactive compounds, and therapeutic cells [32]. Accordingly, in the present study, sodium alginate was used to encapsulate DPP extract in a form of beads.

### 3.1. Zeta Potential and Particle Size Distribution of the Alginate/DPP Extract Solution

**Figure 2:**
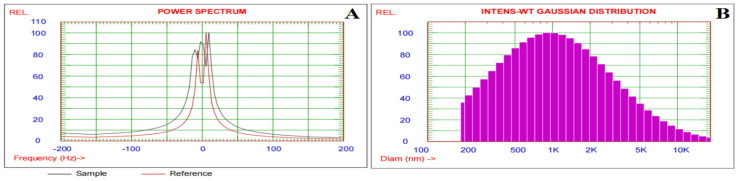
Zeta Potential spectrum (**A**) and size distribution (**B**) of the alginate/DPP extract solution.

Zeta potential and mean hydrodynamic diameter examinations were used to examine the nature and distribution of encapsulated DPP extract in the polymer matrix, as well as the creation of connections between DPP extract and alginate and the results are shown in Figure 2. The sizes and size distributions of alginate and DPP extracts were measured using dynamic light scattering (DLS). Zeta potential measurements were used to evaluate the stability of colloidal systems because they provide a strong indicator of the strength of the connection between colloidal particles. Particles with a zeta potential between −30 and +30 are thought to be extremely stable [33]. The average zeta potential (Figure 2A) was −10.40 mV. The alginate in the solution may have caused the negative zeta potential. The dissociated carboxyl groups of guluronic and mannuronic acids in the alginate molecule are responsible for their negative zeta values [34]. The zeta potential of the alginate/DPP solution indicated strong colloidal stability. The mean particle size in the alginate/DPP solution, as determined by DLS, was 1839.6 nm. As indicated in Figure 2B, eighty percent of the distribution was smaller than 2664.2 nm.

### 3.2. EE % of the Beads

Since it indicates the amount of the original bioactive component trapped in the created beads, encapsulation efficiency can be regarded as the most crucial metric for assessing the effectiveness of the encapsulation procedure. In the current experiment, alginate/DPP extract beads showed an encapsulation efficiency of 94.27 ± 1.47%. Ballesteros et al. [35] stated that the retention capacity of substances within the matrix is typically impacted by the coating material and the encapsulation process. To optimize the incorporation and preservation of the functional substances within the encapsulation matrix, it is crucial to carefully choose both the coating material and the procedure for encapsulation. The entrapment efficiency of the current study may be explained by alginate’s capacity to create hydrogels via ionic crosslinking with divalent cations like Ca^2+^, which makes it ideal for encasing bioactive compounds [36]. The EE % in the current study was higher than that found in El-Kholy et al. [37], who used sodium caseinate and soy lecithin to encapsulate DPP extract. When 20 mg of sodium caseinate was added, the investigation found that the freeze-dried nanocapsules had the highest EE percentage (93.78 ± 3.24%); however, the encapsulation efficiency declined as the caseinate proportion increased.

### 3.3. SEM of the Beads

**Figure 3:**
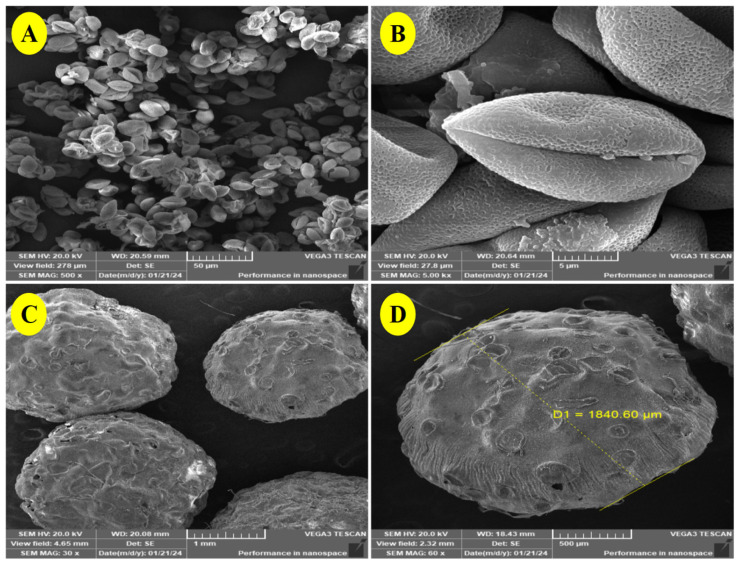
SEM images of DPP grains and alginate/DPP beads. (**A**): SEM image of the whole DPP grains (magnification 500×), (**B**): SEM image of individual DPP grain (magnification 5.00k×), (**C**): SEM image of the alginate/DPP beads (magnification 30×), (**D**): SEM image of the alginate/DPP beads (magnification 60×).

To verify the grain quality, date palm pollen grains were inspected using SEM imaging. As shown in Figure 3A,B, the analysis demonstrated the unique form of pollen grains. The DPP grains were oval, smooth, and reasonably uniform in appearance. They also had a longitudinal groove, which could be a useful plant diagnostic marker [38]. Figure 3C,D depict the form and microstructure of the alginate/DPP extract beads. It has an average size of 1840.60 μm and is spherical in shape. The linkages between DPP extract and alginate resulted in no apparent cracks. A few depressions in the shape of the beads in the SEM imaging were observed, despite the surface’s regularity and smoothness, which are depicted as wet beads in Figure 1. This is because the hydrophilic portion of the polymer matrix contracts during the drying process. Zhou et al. [39] encapsulated *Panax notoginseng* saponin in alginate and *Bletilla striata* polysaccharide, and Reddy et al. [40] encapsulated D-penicillamine drug in karaya gum and sodium alginate microbeads with an average size of 1500 to 1800 μm. These studies provided a representative description of the prepared DPP beads.

### 3.4. HPLC of Beads Extract

**Figure 4:**
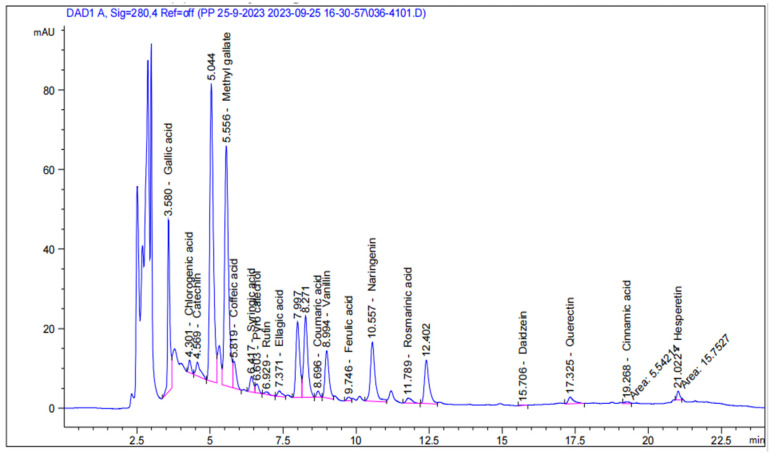
HPLC chromatogram of phenolic profile of the alginate/DPP beads.

The findings (Table 1 and Figure 4) revealed the presence of 18 phenolic compounds (gallic acid, chlorogenic acid, catechin, methyl gallate, caffeic acid, syringic acid, pyro catechol, rutin, ellagic acid, coumaric acid, vanillin, daidzein, quercetin, cinnamic acid, hesperetin, ferulic acid, naringenin and rosmarinic acid) in the DPP beads extract. Methyl gallate, gallic acid, and naringenin were the major phenolics in the extract. In the same context, El- Kholy et al. [37] found 10 phenolic compounds (19.20 μg/mL gallic acid, 191.73 μg/mL catechin, 1.74 μg/mL coffeeic acid, 3.71 μg/mL rutin, 3.91 μg/mL quercetin, 0.46 μg/mL cinnamic acid, 0.56 μg/mL coumaric acid, 0.57 μg/mL ferulic acid, 0.54 μg/mL naringenin, and 0.51 μg/mL propyl gallate) in the ethanol (80%) extract of DPP. For the DPP grown in Egypt, Saudi Arabia, Tunisia, and Algeria, Abdallah et al. [41], Abou Zeid et al. [42], Daoud et al. [43], and Benouamane et al. [44] showed somewhat comparable results, respectively. Numerous biological factors, such as variations in genetics and growing techniques, as well as environmental elements, such as soil conditions, maturation stages, salinity levels, temperature, water availability, and light intensity, can be blamed for this variability [6].

### 3.5. TPC and TFC of Beads Extract

The TPC and TFC of the microsphere extract are presented in Table 2. The phenolic and flavonoid contents of the alginate/DPP microspheres were 66.48 mg GAE/g extract and 15.30 mg CE/g extract, respectively. These TPC and TFC values were greater than those reported by El-Kholy et al. [37], who used sodium caseinate and soy lecithin to encapsulate DPP ethanol extract. Chebout et al. [45] recorded 86.17% and 75.69% encapsulation efficiency for total phenolic and flavonoid, respectively after the encapsulation of phenolic components extracted from Malabar nut (*Justicia adhatoda* L.) leaves by alginate emulsion-gelation methods utilizing response surface methodology. Numerous studies have found varying amounts of phenolic compounds and flavonoids in palm pollen from different sources. Therefore, it is reasonable to ascribe the beads’ phenolic and flavonoid content to the presence of these bioactive components in the palm pollen. According to Daoud et al. [43], TPC was equivalent to 31.93–237.74 mg GAE/g, and TFC was 3.79–75.10 mg QE/g. The TPC was 909.30 mg GAE/100 g DW and 4.31 mg QE/100 g DW, as reported by Sebii et al. [46]. According to El-Kholy et al. [37], the ethanol extract had a TPC of 74.90 mg/g and TFC of 26.28 ± 0.81 mg/g.

### 3.6. In Vitro Antioxidant Activity of the Beads Extract

The ability of natural compounds to donate hydrogen or radical scavenging, is primarily responsible for their antioxidant effects. Antioxidants are required by living organisms to protect against the harmful effects of excessive generation of reactive oxygen species (ROS) and the resulting lipid peroxidation, protein damage, and DNA strand breakage. In the DPPH assay, the color vanishes as hydrogen is converted to DPPH, and the discoloration effect increases with hydrogen transport capacity [47]. Therefore, to evaluate the antioxidant and lipid peroxidation inhibition properties, DPPH and TBARS inhibition tests were carried out. The antioxidant activity of the bead extracts is presented in Table 2. The 50% DPPH inhibition was obtained with 17.33 mg of the alginate/DPP beads extract, and 50% TBARS inhibition was obtained with 9.30 mg extract. Additionally, the alginate/DPP bead extract showed a lower EC_50_ of DPPH than that of encapsulated DPP (19.56 mg) found in the study by El-Kholy et al. [37]. The antioxidant activity of alginate/DPP microspheres is facilitated by the phenolic components of DPP, which function as antioxidants through their reaction with various free radicals. The mechanisms of antioxidant action include chelation of transition metals, sequential proton loss electron transfer, single-electron transfer, and hydrogen atom transfer [48].

### 3.7. Release of Phenolic Compounds from the Beads

The encapsulation approach permits the bioactive substances from the external environment and provides stability in unfavorable conditions until the release of the component is necessary. There are numerous means to trigger the release, including diffusion, degradation, solvents, pH, temperature, and pressure [49]. Table 3 displays the release of phenolic compounds after one, two, and three hours. The current analysis assessed the release mechanism in the buffer. The release of phenolic compounds from the microcapsules depends on the matrix and the solution used. The phenolic release at pH 7.4 (simulated intestinal fluid) was 83.26% greater in the first hour than that at pH 2 (simulated stomach fluid), which was 46.56%. This release also increased with time. At pH 7.4, the maximum release after three hours was 89.81%. This pattern of controlled release can be explained by the high solubility of the alginate at high pH. It is known that the presence of carboxyl groups that dissociate or incorporate protons (H^+^) causes the alginate behavior to be pH-dependent. The increased solubility of alginate at high pH values explains this controlled release pattern. It is well known that alginate behavior is pH-dependent when carboxyl groups that dissolve or incorporate protons (H+) are present. The polymer incorporates protons and becomes insoluble when the pH falls below the pKa range of the constituent acids (pKa of the β-D-mannuronic = 3.38, pKa of the α-L-guluronic = 3.65), protecting the encapsulated molecule. Alginate becomes soluble above this temperature, releasing the active ingredient [50]. This indicates that at pH 2, the phenolic compounds are partially released, and at pH 7.4, they are fully released. These findings align with those of Siles-Sánchez et al. [13], who showed that the majority of phenolic compounds in chitosan are released at higher pH (7.4) and that the release of phenolic compounds increases with time. According to Gorbunova et al. [51], the bioactive inclusions were preserved because the alginate capsules performed well in the hostile stomach environment. The simulated intestinal environment caused the alginate network to soften and swell as desired, allowing the release of bioactive components from the beet green extracts. Harris et al. [52] used spray drying to encapsulate polyphenols in chitosan microparticles. They found that 60% of the polyphenols were liberated from the particles after 4 h at pH 5.7 and between 40 and 45% at pH 6.5. The findings of Fuenmayor and Cosio [53], explained that the bioactive release of phenolics exhibits more significant variations between pH 4.5 and pH 7.0. They also proposed that the cargo-carrier chemical affinity has a significant impact on the release behavior, which is significantly influenced by the pH of the aqueous environment According to Fuenmayor and Cosio [53], the pH-dependent surface charge and pH-dependent ionization of the encapsulated compounds, which undergo significant changes within this pH range have an impact on the release event.

### 3.8. In Vivo Findings

#### 3.8.1. Effect of Alginate/DPP Extract Beads on the Growth Performance

**Figure 5:**
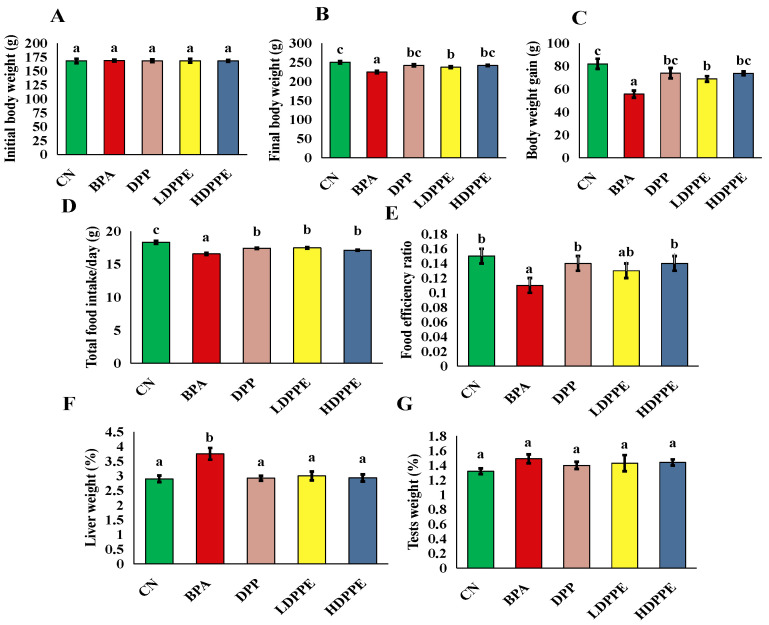
The growth performance parameters. (**A**): the initial weight, (**B**): the final weight, (**C**): the body weight gain, (**D**): food intake/day, (**E**): food efficiency ratio, (**F**): liver weight %, (**G**): testis weight %. On each column, atypical letters denote a significant difference, whereas same letters show a non-significant difference. The mean ± SE is used to describe the data. CN: control normal group, BPA: bisphenol a group, DPP: date palm pollen group, LDPPE: low dose of alginate/DPP beads group, HDPPE: high dose of alginate beads group. Statically (*p* < 0.05), a < ab < b < c.

Body weight is one of the most fundamental metrics for assessing the body’s growth, development, and energy metabolism. Exogenous toxins can induce changes in body weight. In contrast to the CN group, the BPA group showed a significant (*p* < 0.05) decrease in body weight gain, as shown by the data obtained (Figure 5). The BPA group’s liver weight was noticeably (*p* < 0.05) greater than that of the other groups’. The groups’ test weights differed, but not significantly (*p* > 0.05). Additionally, Liu et al. [8] discovered that groups treated with BPA had lower body weights than the control group, indicating that BPA had negative impacts on rats. According to Kobroob et al. [54], BPA could exhibits a catabolic effect. As stated by Liu et al. [8], a typical indicator of toxicological investigations is the liver coefficient, which is the ratio of liver weight to body weight. In line with the pathological outcome, the increase in liver weight and coefficient indirectly represents the liver’s swelling, congestion, and hypertrophy upon exposure to BPA. It is interesting to note that in this study, the groups treated with DPP or the beads experienced a significant (*p* < 0.05) decrease in body weight gain and an increase in relative liver weight. Rats administered a high dose of beads showed body weight gain that was noticeably similar to that of the CN group. It is possible that DPP contains some defensive ingredients that prevent weight loss. These findings are comparable to those of a study by Mohamed et al. [55], who found that administering diabetic rats a 100 mg/kg b.w./day DPP suspension attenuated the reduction in body weight. According to Mohamed et al. [55], this impact can potentially be explained by DPP’s antioxidant properties, which guard against oxidative stress-induced cellular damage.

#### 3.8.2. Effect of Alginate/DPP Extract Beads on the Biochemical Parameters

**Figure 6:**
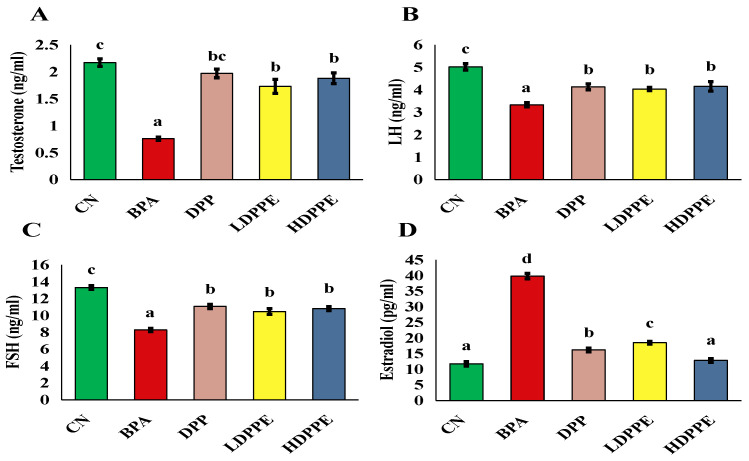
The male fertility-stimulating hormones. (**A**): testosterone, (**B**): LH, (**C**): FSH, and estradiol (**D**). On each column, atypical letters denote a significant difference, whereas the same letters show a non-significant difference. The mean ± SE is used to describe the data. CN: control normal group, BPA: bisphenol a group, DPP: date palm pollen group, LDPPE: low dose of alginate/DPP beads group, HDPPE: high dose of alginate beads group. Statistically (*p* < 0.05), a < b < bc < c.

According to the gonadotropin assessment results (Figure 6), when compared to the control group, the BPA group’s serum levels of LH and FSH were significantly (*p* < 0.05) lower, and their levels of estradiol were significantly (*p* < 0.05) higher, Additionally, rats treated with BPA had significantly (*p* < 0.05) lower testosterone levels than the control group. The fall in LH serum concentration may be directly responsible for the reduction in testosterone production by Leydig cells in BPA-treated mice, as validated by the Wisniewski et al. [56] study. The study of Wisniewski et al. [56], found a dose-dependent decrease in testosterone, FSH, and LH and an increase in serum concentrations of estradiol in BPA-treated animals. Wisniewski et al. [56] declared that exposure to BPA resulted in an imbalance in these hormones, which may have impacted fertility and produced problems in spermatogenesis and sperm maturation. Additionally, Bordbar et al. [10] revealed that rats’ serum levels of testosterone, FSH, and LH decreased in response to both BPA dosages (25 and 50 mg BPA/kg/day). In the current investigation, exposure to either the DPP or both doses of micro-sphere resulted in a significant (*p* < 0.05) drop in estradiol levels and a significant (*p* < 0.05) increase in serum levels of FSH, LH, and testosterone in comparison to the BPA group. According to Salhi et al. [6], the DPP powder’s active constituents of amino acids, fatty acids, and phenolic compounds are responsible for the powder’s improved male hormone impact, while the beads’ effect is mainly due to the phenolic compounds they contain. Mohamed et al. [55] found that when diabetic rats were given DPP aqueous extract (100 mg/kg b.w/day for four weeks), their serum levels of FSH, LH, and testosterone increased. El-Kashlan et al. [57] also found that when male rats with thyroid problems were given DPP ethanolic extract (150 mg/kg b.w/day for 56 days), their serum levels of FSH, LH, and testosterone increased. DPP has been used as a dietary supplement for a long time, especially as a male and female aphrodisiac and fertility enhancer. Both aqueous and ethanolic extracts of DPP showed positive effects on male hormones, which strongly suggests that its polar fraction has considerable biological activity. It is conceivable that these bioactive compounds could influence testosterone, LH, and estradiol levels all of which are crucial for reproductive function by altering hormonal pathways. Additionally, the antioxidant qualities of flavonoids and phenolic substances may help lowering oxidative stress in the reproductive system, which would support better testicular structure and function [6].

**Figure 7:**
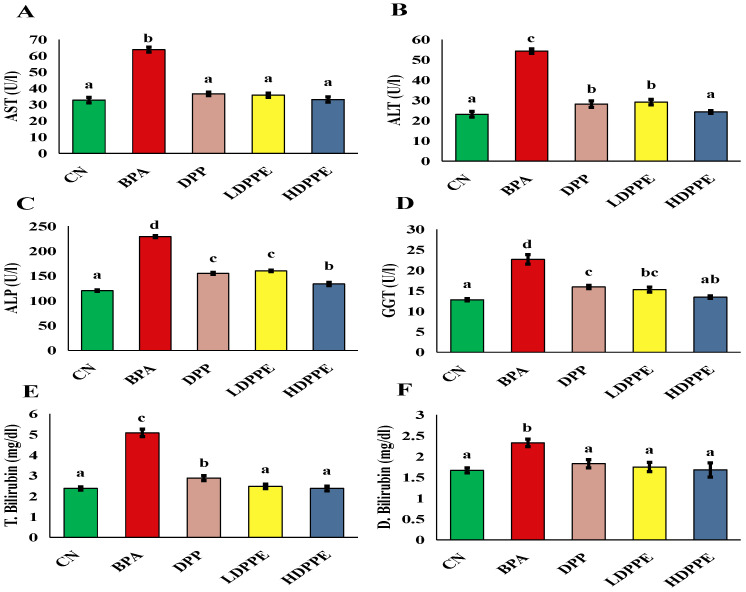
Serum liver markers. (**A**) AST, (**B**) ALT, (**C**) ALP, (**D**) GGT, (**E**) total bilirubin, (**F**) direct bilirubin. On each column, atypical letters denote a significant difference, whereas same letters show a non-significant difference. The mean ± SE is used to describe the data. CN: control normal group, BPA: bisphenol a group, DPP: date palm pollen group, LDPPE: low dose of alginate/DPP beads group, HDPPE: high dose of alginate beads group. Statically (*p* < 0.05), a < ab < b < bc < c < d.

**Figure 8:**
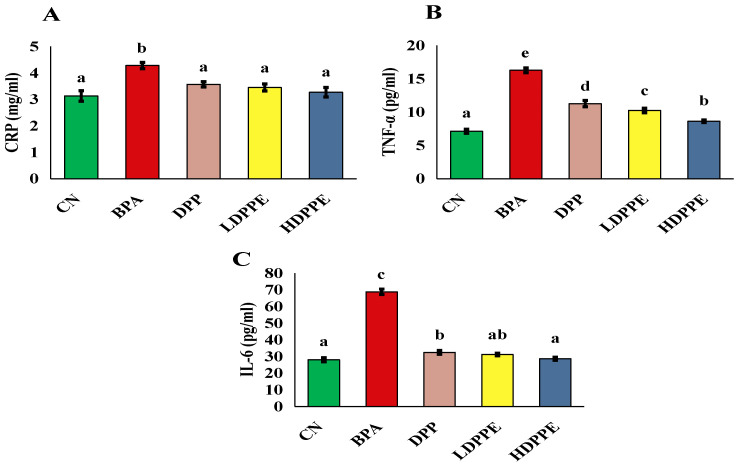
Serum inflammatory cytokine levels: (**A**) CRP, (**B**) TNF-α, (**C**): IL-6. In each column, atypical letters denote a significant difference, whereas the same letters indicate a non-significant difference. The mean ± SE is used to describe the data. CN: control normal group, BPA: bisphenol a group, DPP: date palm pollen group, LDPPE: low dose of alginate/DPP beads group, HDPPE: high dose of alginate beads group. Statically (*p* < 0.05), a < ab < b < c < d < e.

Exposure to BPA causes a number of harmful consequences, such as liver damage, diabetes, infertility, and cancer [8]. Furthermore, the liver is the primary organ responsible for catabolizing foreign substances, making it especially vulnerable to xenobiotic-induced damage [58]. Serum biochemical liver indicators, such as ALP, ALT, AST, GGT, and total and direct bilirubin levels, significantly (*p* < 0.05) increased in the BPA-administered group, as shown in Figure 7. These enzymes are normally absent from the serum; nevertheless, during tissue injury, some of them may leak into the serum. These findings are similar to those of a study by Liu et al. [8], who indicated that exposure to BPA caused hepatotoxicity and disrupted gut microbiota and discovered increases in the biomarkers that are frequently used to represent liver function (AST and ALT). According to Liu et al. [8], inhibiting the SIRT1/PGC-1α pathway and encouraging hepatocyte apoptosis may be the mechanism of liver injury, progressing tissue damage. These alterations in liver markers were substantially (*p* < 0.05) inhibited in animals administered DPP or both microsphere dosages. Similarly, Al-Asmari et al. [5] discovered that administering DPP aqueous suspensions to rats treated with paracetamol at doses of 50 and 100 mg/kg b.w. caused elevations in the serum indicators of liver injury, including bilirubin, AST, ALT, GGT, and ALP. In accordance with Al-Asmari et al. [5], DPP may mediate hepato-protection by a variety of its phytochemicals and bioactive components, including rutin, flavonoids, and phenolic compounds, which have reno-protective and hepato-protective properties as well as antioxidant activity. As BPA exposure causes an inflammatory response, as demonstrated by the current findings (Figure 8), DPP’s anti-inflammatory capability of DPP is another potential mechanism that could contribute to its hepato-protective impact [59].

Liver damage can result from inflammation and oxidative stress [60]. Consequently, this study assessed the markers of oxidative stress and inflammation. Rats in the BPA group had significantly higher (*p* < 0.05) serum levels of TNF-α, IL-6, and CRP than those in the CN group, as seen in Figure 8. These results are consistent with those of a study by Banerjee et al. [61], who found rats exposed to BPA had higher levels of CRP, TNF-α, and IL-6. Conversely, CRP, TNF-α, and IL-6 levels were significantly (*p* < 0.05) decreased in rats exposed to BPA when DPP or both dosages of microsphere were administered. In rats administered doxorubicin, Elblehi et al. [59] investigated the anti-inflammatory properties of DPP ethanolic extract and discovered that it reduced NF-κB p65, TNF-α, IL-1β, and IL-6 levels.

**Figure 9:**
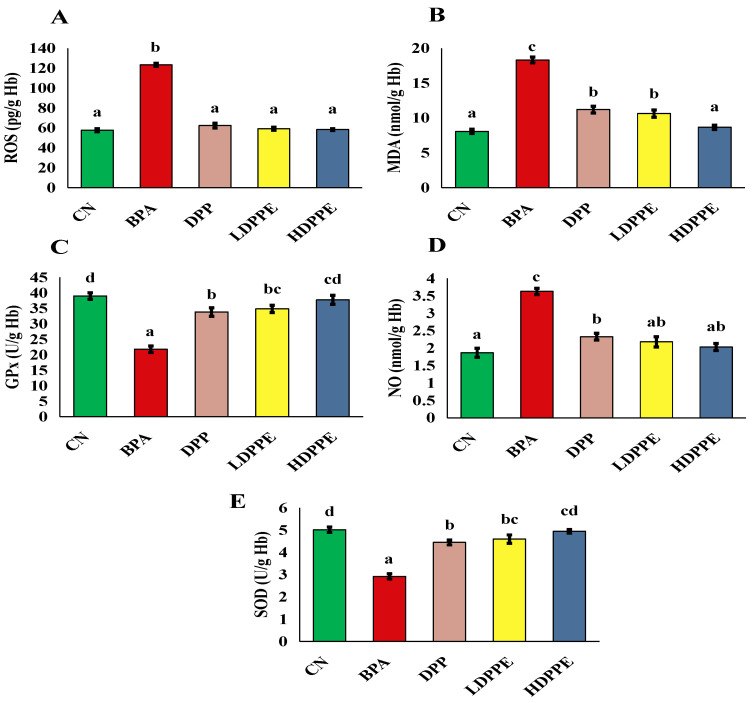
Red blood cell oxidative stress markers. (**A**) ROS, (**B**) MDA, (**C**) GPx, (**D**) NO, (**E**) SOD. On each column, atypical letters denote a significant difference, whereas same letters show a non-significant difference. The mean ± SE is used to describe the data. CN: control normal group, BPA: bisphenol a group, DPP: date palm pollen group, LDPPE: low dose of alginate/DPP beads group, HDPPE: high dose of alginate beads group. Statically (*p* < 0.05), a < ab < b < bc < c < cd < d.

The most common inducer of liver damage is oxidative stress, which reflects an imbalance in the redox system. A reduction in antioxidant defense and an excessive buildup of free radicals, particularly ROS, are the primary causes of oxidative stress, which initiates and encourages liver damage [62]. Excessive ROS accumulation in the body can cause DNA damage, lipid accumulation, and eventually, cell damage or death. Antioxidants can minimize cellular injury caused by the interaction of lipids, proteins, and DNA molecules with ROS [63]. Therefore, oxidative stress caused by environmental contaminants is commonly assessed using antioxidant markers like SOD and GPx. In this study, in contrast to the CN group, the BPA group showed significantly (*p* < 0.05) lower levels of the enzymatic and non-enzymatic defense mechanisms SOD and GPx, and significantly (*p* < 0.05) higher levels of ROS, MDA, and NO, which are indicators of free radical damage, as displayed in Figure 9. In line with the current study’s findings, Maćczak et al. [64] discovered that bisphenols caused oxidative damage in human red blood cells, increasing ROS, MDA, and NO levels, while lowering SOD and GPx levels.

The treatment of BPA-exposed rats with DPP or both dosages of microspheres significantly (*p* < 0.05) reduced ROS, MDA, and NO levels and increased SOD and GPx activity compared to the BPA group. The antioxidant mechanism of polyphenols in either DPP powder or beads may be implicated in their hepato-protective action. Polyphenols, such as gallic, α-coumaric, and ellagic acids, can scavenge free radicals via H-atom transfer, which arises from lipid peroxidation, thereby shielding cell membranes and their contents from oxidative damage [65]. Furthermore, it has been demonstrated that these phenolic compounds improve antioxidant defense via activating the extracellular signal-regulated kinase/nuclear transcription factor–erythroid 2-related factor 2 (ERK/Nrf2) pathways [66].

**Figure 10:**
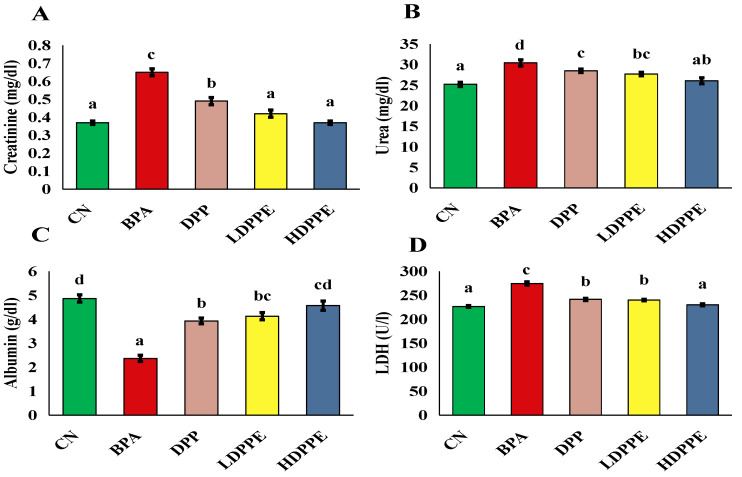
Serum kidney markers. (**A**) creatinine, (**B**) urea, (**C**) albumin, (**D**) LDH. On each column, atypical letters denote a significant difference, whereas same letters show a non-significant difference. The mean ± SE is used to describe the data. CN: control normal group, BPA: bisphenol a group, DPP: date palm pollen group, LDPPE: low dose of alginate/DPP beads group, HDPPE: high dose of alginate beads group. Statically (*p* < 0.05), a < ab < b < bc < c < cd.

As shown in Figure 10, BPA exposure increased serum creatinine and urea levels, while suppressing LDH activity and lowering albumin levels compared to the CN group. Ma et al. [67] explored how bisphenol exposure may result in kidney damage by causing excessive ROS accumulation, oxidative stress, inflammatory reactions, autophagy suppression, and enhanced apoptosis. These processes are interconnected. Oxidative stress can exacerbate damage by directly damaging cells and triggering inflammatory and apoptotic pathways. Rats were treated with 50, 100, and 150 mg/kg, i.p. BPA exposure for five weeks resulted in kidney impairment, as indicated by elevated serum urea and creatinine levels, according to Kobroob et al. [54], who also explained that oxidative stress is a major mediator of BPA-induced nephrotoxicity. According to Priego et al. [68], BPA can cause kidney damage and changes to renal tissue, including tubular dilatation and inflammatory cell infiltration, when administered to healthy C57Bl/6 mice at a dangerous level of 120 mg/kg/day for two and five weeks. These changes in kidney function were avoided in the current investigation by co-administering either DPP or both dosages of beads to the BPA-treated rats. DPP powder and beads may exert renoprotective effects through antioxidant mechanisms. Our results suggest that gallic acid, one of the main phenolic compounds in DPP extract, may have played a role in the renal-protective effect against BPA. This is supported by a study by Saleh et al. [69], who found that gallic acid reduced kidney function and demonstrated a strong protective effect against BPA-induced nephrotoxicity.

#### 3.8.3. Effect of Alginate/DPP Extract Beads on the Histopathological Changes

**Figure 11:**
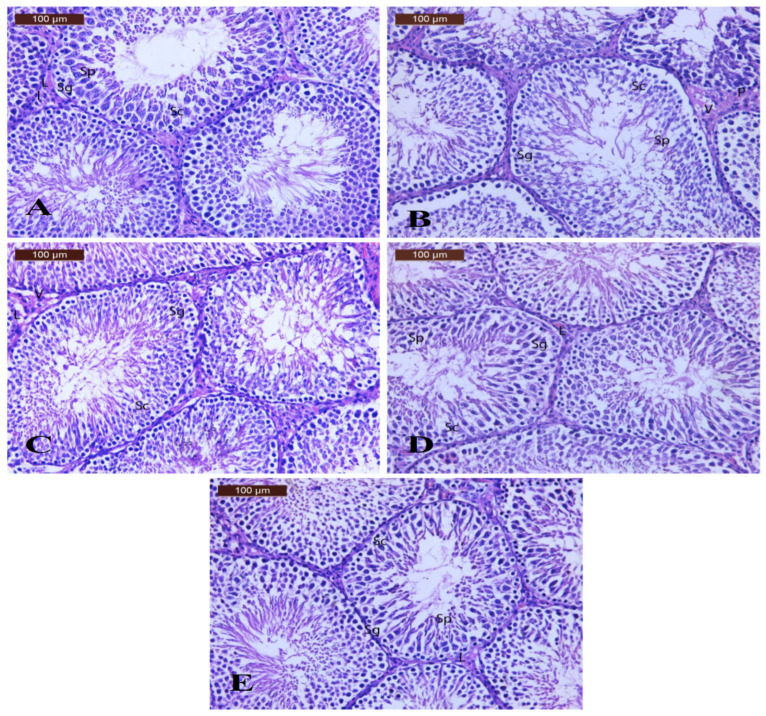
Photomicrographs of rat testis sections of the CN (**A**), BPA (**B**), DPP (**C**), LDPPE (**D**), and HDPPE (**E**) groups.

Regarding the histopathological changes in the testicular tissues (Figure 11), examination of H&E-stained sections of the testes of control rats (Figure 11A) revealed that the parenchyma of the testis was composed of rounded seminiferous tubules. The seminiferous tubules were lined by a stratified germinal epithelium (spermatogonia, spermatocytes, and spermatids) resting on a regular basement membrane. Most of them had narrow lumina. Leydig cells were found in the narrow interstitium. The testis of the BPA group (Figure 11B) showed disturbed architecture of seminiferous tubules, degeneration and exfoliation, germinal epithelium of a seminiferous tubule (Sg, Sc, and Sp), moderately thickened basement membrane, vacuolation, and few pyknotic interstitial cells of Leydig. The testis section of the DPP group (Figure 11C) showed moderate improvement in testicular architecture, with somniferous tubules lined with germinal epithelium (SG), (SC), and (Sp) resting on a thin basement membrane. Other seminiferous tubules showed more or less normal spermatogenic cell lines. Note the slight degeneration of the interstitial tissue and vacuolation (V) with few pyknotic Leydig cells (L). The testis section of the LDPPE group (Figure 11D) showed moderate improvement in testicular architecture, with seminiferous tubules lined with germinal epithelium, spermatogonia (Sg), spermatocytes (Sc), and spermatids (Sp) resting on a thin basement membrane. The other seminiferous tubule is seen with more or less normal spermatogenic cell line. Note the mild, wide interstitial tissue with few Leydig cells. The test section of the HDPPE group (Figure 11E) showed nearly normal testicular architecture, with seminiferous tubules lined with germinal epithelium, spermatogonia (Sg), spermatocytes (Sc), and spermatids (Sp) resting on a thin basement membrane. Note mild wide interstitial tissue with few Leydig cells. According to El-Kashlan et al. [57], pathological alterations in the testicular tissues of rats with hyperthyroidism and hypothyroidism were prevented when administered DPPH ethanolic extract.

**Figure 12:**
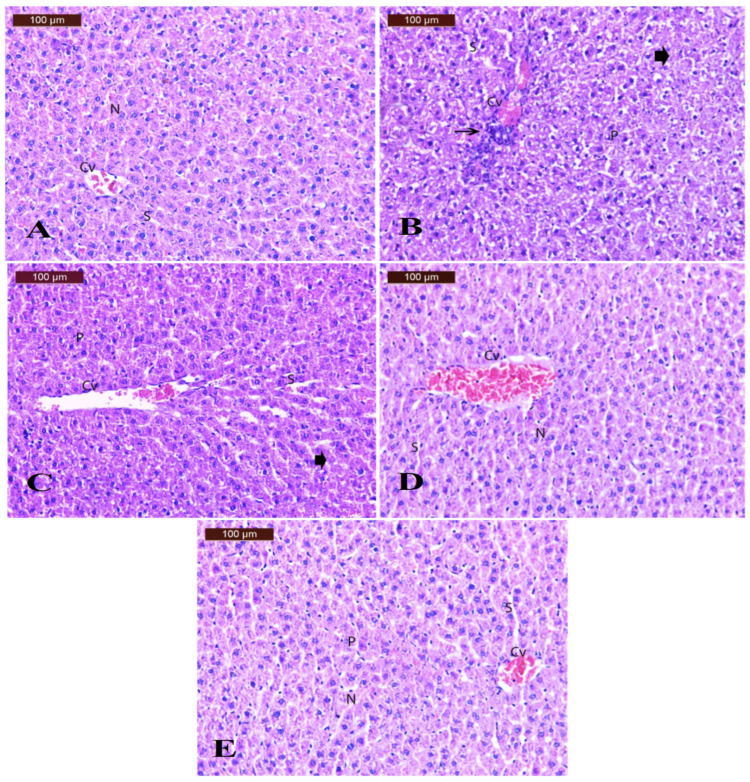
Photomicrographs of rat liver section of CN (**A**), BPA (**B**), DPP (**C**), LDPPE (**D**) and HDPPE (**E**) groups.

Regarding the histopathological changes in the liver tissues (Figure 12), examination of H&E-stained sections of the liver of control rats (Figure 12A) revealed a normal histological picture of the central vein, hepatic sinusoids, and distinct nuclei. The liver of the BPA group (Figure 12B) showed disturbance of the hepatic lobule architecture, congestion of the central vein associated with mild inflammatory cell infiltration, degenerative changes, and pyknotic nuclei. The liver sections of the DPP group (Figure 12C) showed noticeable improvement in liver structure, with more or less normal hepatocytes. Some cells appeared to have pyknotic nuclei (P) with dilated blood sinusoids (S). The liver section of the LDPPE group (Figure 12D) showed moderate ameliorative effects with a congested central vein, moderately dilated blood sinusoids, and nearly normal nuclei. The liver section of the HDPPE group (Figure 12E) showed the hepatic lobule architecture to appear more or less normal with mild congestion of the central vein, mild dilation of blood sinusoids, and few pyknotic nuclei. According to Liu et al. [8], exposure to BPA (30 mg/kg/day by gavage for 30 days) causes damage to the liver tissues, including dysregulation of the hepatic cord, growth of the hepatic sinusoid between the hepatic cords, congestion in the sinusoidal spaces, hepatocyte degeneration, and inflammatory cell infiltration.

## 4. Conclusions

In this study, date palm pollen extract was encapsulated in sodium alginate beads with an efficiency of 94.27%. The phenolic release was maximum at pH 7.4 compared to that at pH 2. The primary phenolic compounds found in the beads were methyl gallate, gallic acid, and naringenin. Alginate/DPP beads significantly protected the liver (as evidenced by decreased liver function parameters), increased levels of male fertility-stimulating hormones, reduced oxidative stress parameters and inflammatory cytokines, and shielded both liver and testicular tissues from BPA-induced damage. Compared to whole pollen, pollen extract beads showed promising results in protecting the liver and improving male sex hormone levels in rats exposed to bisphenol A. This study proposes alginate/DPP beads as a promising functional antioxidant, hepato-protective, and male hormone stimulating nutraceutical.

The most significant limitation of this study was the unethical nature of exposing humans to bisphenol A, as it was conducted on laboratory animals. Therefore, future studies are needed to investigate the sex hormone-enhancing effects of date palm pollen polymeric beads in humans, their thermal stability, and their potential for fortifying certain processed food products.

## Figures and Tables

**Table 1:** Phenolic compounds profile of alginate/DPP beads.

	Conc. (µg/g Extract)
Gallic acid	390.54
Chlorogenic acid	51.35
Catechin	172.15
Methyl gallate	576.00
Coffeic acid	82.34
Syringic acid	50.93
Pyro catechol	59.26
Rutin	23.83
Ellagic acid	30.07
Coumaric acid	7.91
Vanillin	83.29
Ferulic acid	9.05
Naringenin	302.93
Rosmarinic acid	36.43
Daidzein	1.38
Querectin	57.14
Cinnamic acid	2.00
Hesperetin	15.91

**Table 2:** TPC, TFC and antioxidant activity of the beads extract (mean ± SD).

Total phenolic content (mg GAE/g extract)	66.48 ± 0.22
Total flavonoids content (mg CE/g extract)	15.30 ± 0.07
DPPH scavenging activity (EC_50_ value, mg mL^−1^)	17.33 ± 0.11
TBARS inhibition (EC_50_ value, mg mL^−1^)	9.30 ± 0.17

**Table 3:** Phenolic compounds release (%) from the alginate/DPP beads.

pH	1 h	2 h	3 h
2	46.56	57.56	59.43
7.4	83.26	86.29	89.81

## Data Availability

The original contributions presented in this study are included in the article.
